# Exploring the relationship of decentering to health related concepts and cognitive and metacognitive processes in a student sample

**DOI:** 10.1186/s40359-016-0115-6

**Published:** 2016-03-08

**Authors:** Ramona Kessel, Judith Gecht, Thomas Forkmann, Barbara Drueke, Siegfried Gauggel, Verena Mainz

**Affiliations:** Institute of Medical Psychology and Medical Sociology, RWTH Aachen University, Pauwelsstr. 19, 52074 Aachen, Germany

**Keywords:** Decentering, Metacognition, Mindfulness, Attention, Metacognitive monitoring, Self-focussed attention

## Abstract

**Background:**

Decentering, a central change strategy of Mindfulness-Based Cognitive Therapy, is a process of stepping outside of one’s own mental events leading to an objective and non-judging stance towards the self. The study aimed at investigating associated mechanisms of decentering.

**Method:**

The present study investigated the relation of decentering, operationalized by means of the German Version of the Experiences Questionnaire, to severity of depressive symptoms, assessed by the adaptive Rasch-based depression screening, and self-focussed attention, assessed by the Questionnaire of Dysfunctional and Functional Self-Consciousness. Furthermore, the relationship between decentering and a) the ability to shift and allocate attention by means of the Stroop test, and b) metacognitive monitoring, i.e. the absolute difference between judged and real task performance, was investigated. These relationships were examined in 55 healthy students using Pearson’s correlations.

**Results:**

In line with our assumptions, higher decentering scores were significantly associated with lower scores on severity of depressive symptoms, with higher functional- and lower dysfunctional self-focussed attention. Contrary to our expectations, results neither indicated a relationship between decentering and attention ability, nor between decentering and metacognitive monitoring.

**Conclusions:**

The present results suggest that decentering is associated with concepts of mental health (i.e. less severity of depressive symptoms and higher functional self-focussed attention). Overall, the concept decentering seems to be mainly composed of self-focussed aspects when investigated in a healthy sample without intervention. Further investigations of associated concepts of decentering should consider aspects of self-relevance and emotional valence.

## Background

Decentering is described as ‘a process through which one is able to step outside of one’s immediate experience, thereby changing the very nature of that experience’ (Safran and Segal 1990, p. 117). Through this objective observing from a distanced perspective by stepping outside of one’s own mental events, people are enabled to realize that their mental events are no unchangeable truth, but only a constructed reality of the self. This decentered shift in perspective facilitates that a person non-judgmentally accepts the own mental events as what they are, thus as just a thought or an experience. It was examined that the shift in perspective and adaptive stance enhances self-regulation, entails more appropriate reactions to own cognitions, and reduces dysfunctional attitudes towards the own person (Ong et al. 2012; Tanay et al. 2012). The reorientation of attention on thoughts at the present moment, while simultaneously not focussing on its content, is characterized by cognitive flexibility and self-focussed attention (Bishop et al. 2004; Garland et al. 2011; Ortner et al. 2007; Troy et al. 2012). To sum up, the focus of decentering lies on a *shift in perspective*, changing the *relationship* towards the self and inner experiences, leading to a more *objective and non-judging stance* towards the self, and not on changing the particular content of mental events (Ong et al. 2012).

Generally, decentering is viewed as a necessary concept for mental health and a healthy development, whereas the absence of this ability leads to psychological and social dysfunction (Fresco et al. 2007a). Research in healthy individuals suggests that there are habitual interindividual differences in the decentering ability (Feldman et al. 2010; Fresco et al. 2007a; Kahan and Sullivan 2012; Tanay et al. 2012). Although much is known about the beneficial effect of decentering on mental health, the specific processes that are associated with interindividual differences in decentering in healthy subjects remain to be elucidated.

The concept decentering is mainly discussed in the context of mindfulness. There are diverse conceptualisations of mindfulness in the literature (Bishop et al. 2004; Kabat-Zinn 1994; Langer and Moldoveanu 2000), which seem not to be mutually exclusive, but rather overlapping and only differing in focus (for an overview of conceptualizations, see Pagnini and Philips 2015). In an approach by Langer, a mindful state includes an open and new perspective on every novel situation, not relying on prior automatic categories (Langer and Moldoveanu 2000; Pagnini and Philips 2015). Additionally, other researchers postulate that mindfulness means paying attention in the present moment, on purpose and non-judgmentally, which includes non-involvement of emotional assessment (Kabat-Zinn 1994; Pagnini and Philips 2015). Bishop et al. (2004) stress the aspect of attention regulation and an accepting and open orientation on one’s experiences when conceptualizing mindfulness. Generally, being mindful by having an open state of mind is assumed to enhance cognitive flexibility, which implies the ability to interrupt automated responses and rather responding non-habitually (Carson and Langer 2006; Garland et al. 2011; Moore and Malinowski 2009; Troy et al. 2012). This form of flexible information processing is assumed to result in health-related outcomes (Pagnini and Philips 2015). Decentering, as is the concept focussed on in the present study, is known as one central mechanism of change in mindfulness-based cognitive therapy (MBCT) (Feldman et al. 2010; Gecht et al. 2014a; Hick and Chan 2010; Ong et al. 2012; Ortner et al. 2007; Segal et al. 2002; Semple and Burke 2011; Shapiro et al. 2006; Tanay et al. 2012) and has been predominantly investigated in clinical samples. MBCT is one prominent therapy approach within the third wave of cognitive behaviour therapy (CBT; Hayes 2004). Several studies revealed that MBCT is effective in preventing depressive relapse for remitted patients (for a review see Fjorback et al. 2011), and in reducing symptoms of currently depressed patients (Barnhofer et al. 2009; Kenny and Williams 2007; Kingston et al. 2007; Van Aalderen et al. 2012). Additionally, several studies demonstrated the effectiveness of mindfulness-based interventions for the reduction of symptoms in diverse physical and mental health problems in clinical as well as non-clinical samples (for reviews see Grossman et al. 2004; Keng et al. 2011). It has been found that patients’ decentering ability can be enhanced through CBT and MBCT (Bieling et al. 2012; Carmody et al. 2009; Fresco et al. 2007b; Fresco et al. 2011; Hick and Chan 2010; Segal et al. 2002; Teasdale et al. 2002). Furthermore, it could be demonstrated that depressive patients have a lower decentering ability than healthy control subjects (Teasdale et al. 2002), and that decentering is negatively associated with depressive patients’ relapse rate after therapy (Fresco et al. 2007b; Teasdale et al. 2002).

Interestingly, the influence of mindfulness-based interventions on cognitive processing was experimentally examined (e.g. Alberts and Thewissen 2011; Anderson et al. 2007; Chambers et al. 2008; Jha et al. 2007; Ortner et al. 2007; Van den Hurk et al. 2012; Wenk-Sormaz 2005; for a review see Van der Velden et al. 2015), whereas, to our knowledge, investigations of specific psychological mechanisms underlying decentering remain spare. This is surprising since decentering is regarded as a central mechanism of change in psychotherapy. Hence, it would be of great practical importance to clarify the psychological correlates of interindividual differences in decentering. Knowledge about mechanisms associated with interindividual differences in decentering would be important for the development of psychotherapy or health interventions that would train these special processes to further increase therapy outcome and a healthy functioning.

The conceptualization of decentering as a metacognitive strategy (e.g. see Bernstein et al. 2015; Garland et al. 2011; Lebois et al. 2015; Troy et al. 2012) allows to focus on the following aspects within decentering that might vary interindividually: In a decentered state people are ought to be able to a) allocate attention on own mental events, while b) simultaneously only *observing* and not focussing on its content (Bishop et al. 2004; Garland et al. 2011; Ortner et al. 2007; Troy et al. 2012).

First, a) cognitive resources like the ability to shift and allocate attention appear to be an important prerequisite for decentering. Lutz et al. (2008) reported that through focussed attention meditation, as used in MBCT, peoples’ sustained and selective attention could be enhanced. Furthermore, studies revealed that the inhibition of automatic responses is enhanced by meditation. It could be demonstrated that meditators, in comparison to non-meditators or participants receiving no meditation practice, showed a reduction in habitual responding on the Stroop test, i.e. reacted with less interference when trying to prevent an automated response of reading words instead of ignoring word content, but only naming the colour of written words (for a detailed task description see e.g. Moore and Malinowski 2009 or the method section of the present paper) (Moore and Malinowski 2009; Wenk-Sormaz 2005). Ortner et al. (2007) found that people with experience in mindfulness meditation showed a reduced interference effect on the emotional interference task. Moreover, Lebois et al. (2015) investigated that decentering ability was enhanced by mindful attention intervention.

Second, after allocating attention to one’s own thoughts, by definition decentered people are ought to have b) metacognitive abilities to *observe*, respectively monitor these thoughts purposefully and non-judgmentally. In this respect, it can be assumed that decentering and metacognitive monitoring might be associated. Metacognitive monitoring is defined as the subjective assessment of one’s own cognitions and knowledge, represented by information flow from a lower object-level to a higher meta-level necessary for adapting behaviour (Koriat and Shitzer-Reichert 2002; Nelson and Narens 1990). Specifically, researchers postulate that metacognitive monitoring processes of own thoughts lead to a decentered perspective (Allen et al. 2006; Garland et al. 2011; Segal et al. 2002, 2013). As it is known that depressive patients are impaired in their decentering ability, some support for the above described assumption that decentering might be related to metacognitive monitoring can additionally be derived from research findings on depressive patients. Depressed people showed impaired metacognitive monitoring abilities compared to partially remitted patients and control subjects (Sheppard and Teasdale 2004; Slife and Weaver 1992). Metacognitive monitoring ability is typically assessed by means of metacognitive judgments, in which people are asked to judge their own cognitive performance (judgment of performance, JOP) (for an overview see Koriat 2007). Subsequently, these JOPs are compared to the real task performance by means of the absolute difference between these two scores (e.g. Slife and Weaver 1992).

The present study aimed at investigating the relation of decentering to severity of depressive symptoms, self-focussed attention, as well as the ability to shift and allocate attention and metacognitive monitoring in a sample of healthy subjects. We hypothesized that a higher decentering ability will be associated with less severe depressive symptoms and with lower dysfunctional and higher functional self-focussed attention. Furthermore, we hypothesized that people scoring higher on decentering will also show a higher ability to shift and allocate attention and a higher metacognitive monitoring ability. In addition, it is assumable that variance in decentering among healthy participants is smaller than in previous studies comparing healthy controls with depressed patients. Therefore, we hypothesized that analyses of low and high decentering groups would probably indicate hidden effects of the above hypothesized associated processes.

## Method

### Participants

The sample of the study consisted of 55 healthy students from RWTH Aachen University, who did not suffer from any physical or mental illness. Participants’ mean age was 24 ± 3 years (range 18–32) and most of them were female (69 %). The majority of participants were medical students (35 %), followed by psychology (20 %) and engineering (18 %). Exclusion criteria for participation were suffering from mental illnesses, insufficient command of the German language, colour vision deficiency, and dyslexia. All participants received a financial compensation for their participation. Approval for the study was provided by the ethics committee of the medical faculty of the RWTH Aachen University (EK148/11).

### Material

#### Decentering

As a measure of decentering the EQ-D (Gecht et al. 2014b) was used. The EQ-D is a German version of the Experiences Questionnaire (EQ; Fresco et al. 2007a). The EQ-D consists of 8 questions assessed on a 5-point Likert scale (0 = *never*, 4 = *always*). The questionnaire includes two subscales consisting of four items each. Consequently, for each subscale scores can range from 0 to 16. One subscale represents the decentering aspect of ‘accepting self-perception’ (ASP) (e.g. ‘I can accept myself as I am’). The other subscale represents the decentering aspect of ‘distanced perspective’ (DP) (e.g. ‘I can separate myself from my thoughts and feelings’). The four items of each of the two subscales were combined and summed up into a single index for each subscale (ASP: Cronbach’s α = 0.70; DP: Cronbach’s α = 0.70).^1^ Higher scores indicated a higher ability of the respective aspect of decentering. Psychometric analyses of the EQ-D by Gecht et al. (2014b) revealed adequate construct validity. Note that the full 20-item-version of the original EQ (Fresco et al. 2007a) was administered as it is recommended by Gecht et al. (2014b).

#### Depressive symptoms

As a measure of severity of depressive symptoms, the adaptive Rasch-based depression screening (A-DESC) was used (Forkmann et al. 2009; Forkmann et al. 2013). The A-DESC is a well-validated instrument to assess the severity of depressive symptoms and may also be used as a screening tool by applying the cut-off scores provided (Forkmann et al. 2009; Forkmann et al. 2013). Participants were asked to answer 36 items on a 5-point Likert scale (0 = *never*, 4 = *always*), Cronbach’s α = 0.94^1^. Lower scores indicated less severity of depressive symptoms. The A-DESC showed adequate criterion validity (Forkmann et al. 2013).

#### Self-focussed attention

As a measure of self-focussed attention, the Questionnaire of Dysfunctional and Functional Self-Consciousness (DFS; Hoyer 2000) was used. This questionnaire includes one subscale measuring dysfunctional self-focussed attention, consisting of 14 items (e.g. ‘Once I start thinking about a problem I cannot stop easily’), Cronbach’s α = 0.91^1^. The other subscale of the questionnaire measures functional self-focussed attention, consisting of eight items (e.g. ‘I am confident of being able to solve a personal problem, even if there is no solution in sight at the beginning’), Cronbach’s α = 0.77^1^. Each item is assessed on a 5-point Likert scale (0 = *absolutely not applicable*, 4 = *absolutely applicable*). The DFS showed adequate psychometric properties (Hoyer 2000).

#### Attention task

As a measure of shifting and allocating attention, the German version of the Stroop test was used (Bäumler 1985). This test assesses selective and executive attention by measuring inhibitory processes. This task was administered in form of a paper-and-pencil test using a stopwatch. Participants are asked to name colours while simultaneously suppressing automatic reading processes, which requires cognitive flexibility. The test consisted of three different task types of increasing difficulty. First, participants had to read the words “red”, “green”, “yellow” and “blue” written in black ink (Colour Word Reading, CWR). Second, participants had to name the colour of control patches, which means for example naming “yellow” when a yellow patch is presented (Colour Patches Naming, CPN). In this task type there were no written words, only colour patches. Third, participants had to name the incongruent colour of colour words, for example naming red when the word ‘green’ was written in red ink (Interference, INT). Participants were instructed to read the words or name the colours as fast and accurate as possible. Participants had to perform three trials. Completion time (time from naming the first item until naming the last one of each page, respectively each subtask) was recorded in seconds with a stopwatch. Interference refers to the decrement in performance for the incongruent task (INT) in comparison to only naming colours, and is calculated as the difference in reaction time between INT and CPN (MacLeod 1991). The higher the difference between the two tasks is, the higher is the interference and the lower the ability to shift and allocate attention. For the purpose of the present study, the mean reaction time of INT as well as mean Interference was included in the analyses. For further details on this method, see Kessel et al. (2014).

#### Metacognitive monitoring

Participants’ ability to monitor their own performance in the attention task was assessed by metacognitive judgments of performance (JOPs). Participants were asked to judge after the subtasks of the Stroop test the time (in seconds) they needed to perform the task (completion time). As index for the metacognitive monitoring ability, the absolute difference between judged and real performance was calculated, representing absolute monitoring accuracy (Mengelkamp and Bannert 2009). The absolute difference score is a common measure used for assessing absolute judgment accuracy respectively congruence between these two values and represents the magnitude of judgment error from the true score (see e.g. Edwards 1994; Holmbeck et al. 2002; Mengelkamp and Bannert 2009; Schraw and Roedel 1994). The smaller this difference is, the higher is the accuracy respectively the monitoring ability. In order to ensure that the judgments were based on internal monitoring processes, no feedback of task performance was provided. For further details on this method, see Kessel et al. (2014). For the purpose of the present study, the mean absolute differences between judged and real task performance regarding completion time of INT were included in the analyses, as this is the measure used for the assessment of the ability to shift and allocate attention.

### Procedure

Participants were recruited via notices in different departments of the university. A telephone interview was conducted before the individual examination in order to check exclusion criteria, and to acquire general demographic information. At the day of the examination in the laboratory, participants were given general information about the experimental procedure, and they provided written informed consent. Then, a clinical screening interview based on the International Diagnostic Checklist (ICDL; Hiller et al. 1997) for depression was conducted in order to check for absence of a depressive disorder. After this, participants were asked to fill in the questionnaires and to conduct the attention task, including the JOPs after each subtask.

### Statistical analysis

All data was analyzed in SPSS 20.0. Adequate sample size was calculated with G*Power 3.1 (Faul et al. 2007). An a priori power analysis for t-tests/Correlation/two-tailed was conducted with the following parameters: Effect size *ρ* = 0.35,^2^ α = 0.05, power = 0.80.

For testing the research hypotheses, Pearson’s correlations *r* were used. According to Cohen’s (1988) guidelines, a Pearson’s correlations *r* of 0.1 represents a small effect, 0.3 represents a medium effect, and 0.5 represents a large effect. Because of rather small variance on decentering scores for the present sample, an Extreme Groups Approach (Preacher et al. 2005) was applied additionally. Participants were split up into three decentering groups by means of tertile split for each decentering subscale separately. After this, only the lowest and the highest tertiles, representing people with either ‘low’ or ‘high’ scores on decentering, were included in further analyses. For these analyses of low and high decentering groups, independent samples *t*-tests were conducted, investigating the differences of the respective variables between these low and high decentering groups. For these analyses, Effect sizes (ES) were calculated according to Cohen’s d (1988) and corrected by means of Hedges and Olkins’ formula (1985). ES of 0.2 to 0.5 represent a small effect, ES of 0.5 to 0.8 represent a medium effect and ES above 0.8 represent a large effect.

## Results

### Relation of decentering to severity of depressive symptoms and self-focussed attention

Means and standard deviations of the respective variables and results of the Pearson’s correlations *r* investigating the relationship between decentering, depressive symptoms, and dysfunctional and functional self-focussed attention are presented in Table 1. All correlations were significant (*p* < 0.05).

**Table 1:** Means (*M*), standard deviations (*SD*), and correlations representing the relationship between decentering, severity of depressive symptoms and self-focussed attention

	*M* (*SD*)	2	3	4	5
1 Accepting self-perception^a^	12.6 (2.1)	.31*	−.51**	−.43**	.40**
2 Distanced perspective^a^	9.3 (2.6)	–	−.41**	−.64**	.39**
3 Depressive symptoms^b^	−2.4 (0.8)	–	–	.61**	−.28*
4 Dysfunctional self-focussed attention^c^	33.5 (8.4)	–	–	–	−.31*
5 Functional self-focussed attention^c^	29.9 (4.2)	–	–	–	–

*p < 0.05

**p < 0.01

^a^assessed with the German version of the Experiences Questionnaire (EQ-D)

^b^assessed with the adaptive Rasch-based depression screening (A-DESC)

^c^assessed with the Questionnaire of Dysfunctional and Functional Self-Consciousness (DFS)

Both decentering subscales, i.e. ASP and DP, showed significant negative correlations with depressive symptoms. This indicates that the higher participants scored on the decentering measures, the lower they scored on the measure of depressive symptoms. ASP and DP showed significant negative correlations with dysfunctional self-focussed attention and significant positive correlations with functional self-focussed attention. This indicates that participants scoring higher on the decentering measures reported higher functional and lower dysfunctional self-focussed attention.

### Relationship between decentering and the ability to shift and allocate attention

Means and standard deviations of the respective variables and results of the Pearson’s correlations *r* investigating the relationship between decentering and the two measures for the ability to shift and allocate attention (INT and Interference) are presented in Table 2. None of these correlations reached significance (*p >* 0.05).

**Table 2:** Means (*M*), standard deviations (*SD*), and correlations representing the relationship between decentering and attention ability

	*M* (*SD*)	2	3	4
1 Accepting self-perception^a^	12.6 (2.1)	.31*	−.13	−.09
2 Distanced perspective^a^	9.3 (2.6)	–	.16	.23
3 Interference task (INT)	56.5 (8.8)	–	–	.78**
4 Interference^b^	17.0 (5.6)	–	–	–

*p < 0.05

**p < 0.01

^a^assessed with the German version of the Experiences Questionnaire (EQ-D)

^b^difference in reaction time between the Stroop tasks Interference (INT) and Colour Patches Naming (CPN)

### Relationship between decentering and metacognitive monitoring ability

Means and standard deviations of the respective variables and results of the Pearson’s correlations *r* investigating the relationship between decentering and metacognitive monitoring ability are presented in Table 3. None of these correlations reached significance (*p >* 0.05).

**Table 3:** Means (*M*), standard deviations (*SD*), and correlations representing the relationship between decentering and metacognitive monitoring ability

	*M* (*SD*)	2	3
1 Accepting self-perception^a^	12.6 (2.1)	.31*	.08
2 Distanced perspective^a^	9.3 (2.6)	–	.12
3 Monitoring ability^b^	19.9 (17.5)	–	–

*p < 0.05

^a^assessed with the German version of the Experiences Questionnaire (EQ-D)

^b^indexed as the absolute difference between judged and real Interference task performance (INT)

### Analyses of low and high decentering groups

Group sizes, means, and standard deviations of the three tertiles are presented in Table 4.

**Table 4:** Group sizes (*N*), means (*M*), and standard deviations (*SD*) of tertile split^a^ on decentering subscales^b^

		*N*	*M*	*SD*
ASP^c^	group 1	18	10.4	1.2
	group 2	19	12.6	0.7
	group 3	18	14.9	0.9
DP^d^	group 1	18	6.3	1.5
	group 2	19	9.4	0.7
	group 3	18	12.0	1.5

^a^group 1 = tertile with low decentering scores; group 2 = tertile with medium decentering scores; group 3 = tertile with high decentering scores

^b^assessed with the German version of the Experiences Questionnaire (EQ-D)

^c^subscale accepting self-perception

^d^subscale distanced perspective

Results of the *t*-tests exploring whether there is a significant difference in the ability to shift and allocate attention between the low and high decentering groups are presented in Table 5. Means of the two groups did not differ significantly concerning participants’ ability to shift and allocate attention (*p >* 0.05).

**Table 5:** Group sizes (*N*), means (*M*), standard deviations (*SD*), and results of the t-tests representing the difference in the ability to shift and allocate attention between people with low (group 1) and high (group 3) scores^a^ on decentering based on tertile split of the two decentering subscales^b^, i.e. accepting self-perception (ASP) and distanced perspective (DP)

			*N*	*M*	*SD*	t	p	df	ES
Tertile split ASP	Interference task	group 1	18	57.1	9.2	.95	.35	34	0.30
		group 3	18	54.6	6.9				
	Interference^c^	group 1	18	17.1	6.3	.42	.68	34	0.14
		group 3	18	16.3	4.8				
Tertile split DP	Interference task	group 1	18	55.6	6.8	−.64	.52	34	−0.20
		group 3	18	57.3	9.4				
	Interference^c^	group 1	18	15.2	3.1	−1.37	.18	34	−0.45
		group 3	18	17.1	5.0				

^a^group 1 = tertile with low decentering scores; group 3 = tertile with high decentering scores

^b^assessed with the German version of the Experiences Questionnaire (EQ-D)

^c^difference in reaction time between Stroop tasks Interference (INT) and Colour Patches Naming (CPN)

Results of the *t*-tests exploring whether there is a significant difference in metacognitive monitoring ability between the low and high decentering groups are presented in Table 6. Means of the two groups did not significantly differ concerning participants’ metacognitive monitoring ability (*p >* 0.05).

**Table 6:** Group sizes (*N*), means (*M*), standard deviations (*SD*), and results of the t-tests representing the difference in metacognitive monitoring ability^a^ between people with low (group 1) and high (group 3) scores^b^ on decentering based on tertile split of the two decentering subscales^c^, i.e. accepting self-perception (ASP) and distanced perspective (DP)

			*N*	*M*	*SD*	t	p	df	ES
Tertile split ASP	Monitoring ability	Group 1	18	16.3	10.3	−.79	.43	34	−0.26
		Group 3	18	19.9	15.8				
Tertile split DP	Monitoring ability	Group 1	18	15.4	8.7	−1.17	.25	34	−0.38
		Group 3	18	22.1	23.0				

^a^indexed as the absolute difference between judged and real Interference task performance (INT)

^b^group 1 = tertile with low decentering scores; group 3 = tertile with high decentering scores

^c^assessed with the German version of the Experiences Questionnaire (EQ-D)

## Discussion

The aim of the present study was to investigate the relation of decentering to severity of depressive symptoms, self-focussed attention, as well as the ability to shift and allocate attention and metacognitive monitoring in a sample of healthy subjects. In line with our assumptions, decentering was significantly associated with severity of depressive symptoms and self-focussed attention. Contrary to our expectations, results neither indicated a relationship between decentering and attention ability, nor between decentering and metacognitive monitoring ability. Results of low and high decentering group analyses revealed similar findings. In the following sections, we will discuss our findings.

### Relation of decentering to severity of depressive symptoms and self-focussed attention

We hypothesized that a higher decentering ability would be associated with less severe depressive symptoms, and with lower dysfunctional and higher functional self-focussed attention. Our results confirmed this hypothesis. As it can be assumed that less severity of depressive symptoms and high functional self-focussed attention are linked to mental health in general, our finding may suggest that decentering is accompanied by general mental health (e.g. Fresco et al. 2007a). Importantly, in the present study relationships between decentering and depressive symptoms emerged that are similar to results in prior investigations with healthy samples (e.g. Gecht et al. 2014a, b).

### Decentering and attention

Pursuing considerations derived from the conceptualisation of decentering and based on research findings within this field (e.g. Jha et al. 2007; Lutz et al. 2008; Moore and Malinowski 2009), it was hypothesized that people with higher decentering abilities would also show a higher ability to shift and allocate attention. Against expectations, results indicated that decentering was not significantly associated with both of the acquired attention indices. Instead, the present results are in line with Anderson et al. (2007) and Van den Hurk et al. (2012), who could not find a relation between mindfulness and diverse attention processes, amongst others measured by means of the Stroop test. These researchers argue that *awareness* instead of attention, respectively a shift in attitude towards an open and accepting stance (according to one central component of mindfulness by Bishop et al. (2004)) represents the central aspect of mindfulness. As decentering is viewed as a central key mechanism facilitating a mindful state (Feldman et al. 2010; Gecht et al. 2014a; Hick and Chan 2010; Ong et al. 2012; Ortner et al. 2007; Segal et al. 2002; Semple and Burke 2011; Shapiro et al. 2006; Tanay et al. 2012), it is assumable that these findings are attributable to decentering. Overall, it appears that in the present investigation the assumed effects of decentering might mainly be driven by an aware state of mind and an accepting stance towards inner mental events of the self rather than the ability to shift and allocate attention.

In sum, the present results on the relationship between decentering and attention lead to two possible conclusions. The first possibility is that decentering and attention are rather distinct and unrelated concepts. The other possibility is that the association between decentering and attention performance only becomes evident in the aspect of awareness and accepting stance towards own mental events. This needs to be further clarified using additional tasks focussing on attention performances that are affected by self-relevant and emotionally valent stimuli.

### Decentering and metacognitive monitoring

As by definition decentered people are ought to have metacognitive abilities enabling them to monitor their thoughts purposefully and non-judgmentally, it was hypothesized that people scoring higher on decentering would have a higher metacognitive monitoring ability. Results indicated no significant relationship between decentering and metacognitive monitoring ability.

As a possible explanation for this finding, it can be speculated that data did not reveal any association between decentering and metacognitive monitoring ability, because metacognitive monitoring as assessed in the present task implicitly included some performance evaluation and not just *observing* the own performance in a decentered way. Nelson and Narens’ (1990) postulate in their metacognitive framework, that metacognitive monitoring is always linked with control processes in order to adapt behaviour. Decentering, however, does not comprise evaluative or adaptive processes. Possibly, as the associations between decentering and metacognitive monitoring were not evident in our study, task performance might have predominantly triggered control processes, which in turn could have covered the assumed associations between decentering and monitoring ability.

Additionally, the distinction between metacognitive insight and metacognitive knowledge made by Teasdale (1999) in his Interacting Cognitive Subsystems framework (ICS) can serve as a more refined perspective on how decentering could relate to monitoring abilities. Metacognitive insight, i.e. emotionally *experiencing* that thoughts are not facts, is understood as a higher order mechanism acting complementary to metacognitive knowledge, i.e. just factually *knowing* that thoughts are not facts. It is postulated that a decentered perspective or experiencing mode is a form of metacognitive insight (Allen et al. 2006; Teasdale et al. 2002). Metacognitive monitoring of cognitive performance as assessed by the present task could have predominantly triggered a form of factual metacognitive knowledge instead of representing metacognitive insight. Therefore, our results further suggest that decentering could rather resemble the aspect of emotional *experience* of the fact that own mental events are not reality, thus a metacognitive *insight* mode. This might again indicate that decentering involves being aware while monitoring *self-referential emotional* aspects.

Similar to the relationship between decentering and attention, two possible conclusions can be drawn from our results regarding the association of decentering and metacognitive monitoring. The first is that decentering and metacognitive monitoring are unrelated concepts. The second possibility is that the association between decentering and metacognitive monitoring becomes evident in the aspect of self-referential emotional valence, as postulated by Teasdale et al. (1999, 2002) in the concept of decentering as metacognitive insight, thus an emotionally experiencing mode. This aspect needs further clarification using experimental tasks in which monitoring of self-referential emotionally valent mental events would be assessed.

### General discussion, strengths, and limitations

Having discussed our results in detail above, some general aspects remain to be mentioned that might have contributed to the interesting but unexpected findings. Most generally speaking, decentering is of a complex nature and definitions differ with emphasis on different components that are in the focus of research interest (Fresco et al. 2007a; Safran and Segal 1990). In the present case, decentering ability, assessed by means of the EQ-D, focussed on two aspects, which were the accepting self-perception (ASP) and the distanced perspective (DP). As such, decentering was neither significantly associated with the here acquired attentional nor the metacognitive monitoring processes. Overall, it seems that the aspect of an objective stance towards *the self* constitutes the central aspect of decentering. Therefore, decentering was rather related to processes like self-focussed attention. One major strength of the present study is the successful operationalization of decentering by means of the EQ-D. We could show comparable variance of EQ-D items in the present non-clinical sample to other studies assessing decentering by means of the EQ in non-clinical samples (Fresco et al. 2007a; Tanay et al. 2012).

Finally, some limitations have to be mentioned that may be considered in future studies. The present study was conducted with cross-sectional data acquired from a non-clinical sample and without any manipulating intervention, so no causal inferences should be drawn. Generally, a larger sample and an investigation in different, also clinical samples with more variance on decentering would be beneficial to further investigate possible mechanisms associated with decentering ability. Furthermore, as the EQ was originally designed to measure therapeutically induced changes (Fresco et al. 2007a), it could be that items appeared rather unfamiliar to the investigated healthy student sample (Gecht et al. 2014b), e.g. ‘I can actually see that I am not my thoughts’. This could have interacted with the likelihood of the participants to agree to an item or not.

## Conclusion and future directions

The present study is a first contribution to the investigation of possible mechanisms associated with decentering. Results revealed that a higher decentering ability, operationalized by means of the EQ-D, was related to less severe depressive symptoms, higher functional and lower dysfunctional self-focussed attention. As it can be assumed that these concepts are linked to general mental health, our finding suggests that a higher decentering ability is accompanied by general mental health (Fresco et al. 2007a).

Unexpectedly, decentering was neither significantly associated with the assessed attentional processes, nor related to the here acquired metacognitive monitoring abilities. Therefore, results suggest that decentering and ability to shift and allocate attention as well as metacognitive monitoring are not associated, at least as it is operationalized in the present study. In conclusion, it seemed that decentering is principally constituted by self-focussed aspects highlighting its potential role within the acquisition of a non-judging and objective stance towards *the self*.

Future research is needed to distinguish and clarify the underlying processes of decentering, and to further establish its role in relation to concepts like metacognition and cognitive abilities, as well as considering other concepts. A starting point would be stronger consideration of the relevance of self-referential processes for decentering. To focus on the non-judgmental and accepting stance towards the self could offer further insight into whether this aspect may be an important aspect of decentering. In order to further clarify its relation to cognitive and metacognitive abilities, self-relevant autobiographical stimuli within experimental tasks investigating these two processes could be reasonable to gather more information about the self-focussed aspect and its emotional valence of decentering in relation to attention and monitoring abilities. Finally, once having more clarity about the central mechanisms of the concept decentering, investigating and manipulating the degree of a person’s decentering ability by brief interventions would elucidate whether its underlying processes can be trained, leading to improvements in decentering and on the long term to improving mental health.

## Footnotes

1 Note that these Cronbach’s α values were calculated on data of the present sample

2 Note that in the light of previous studies which usually found effect sizes > .4 (e.g. Fresco et al. 2007a, b; Gecht et al. 2014a, b; Ortner et al. 2007) smaller effects were expected here, resulting in a slightly more conservative power analysis
